# Smartphone Sensor Dataset for Driver Behavior Analysis

**DOI:** 10.1016/j.dib.2022.107992

**Published:** 2022-02-25

**Authors:** Pawan Wawage, Yogesh Deshpande

**Affiliations:** Vishwakarma University*,* India

**Keywords:** Driver behavior analysis, Machine learning, Smartphone sensor dataset, DB classification

## Abstract

Driving is considered one of the most difficult tasks because the driver is responsible for a variety of other responsibilities in addition to driving. The primary responsibility of a driver should be to properly operate a vehicle while concentrating solely on driving. However, he/she must also complete various secondary jobs at the same time. Modeling realistic driving behavior proved tough for researchers and scientists. With this goal in mind, we constructed a Smartphone sensor dataset of Indian drivers, complete with driving parameters that have a significant impact on driving behavior. As a result, we created a dataset using Smartphone sensors such as the accelerometer and gyroscope. The data is organized into day-by-day folders, each with seven subfolders. We are confident that the suggested dataset will be beneficial in the training, testing, and validation of a machine learning model for driver behavior classification or reorganization.

## Specifications Table

**Table:** 

Subject	*Machine Learning, Driver Behavior Classification, Human-Machine Interaction.*
Specific subject area	*Driver Behavior Classification with Smartphone sensor data.*
Type of data	*Smartphone Sensor (Accelerometer, Gyroscope, etc.) Data.*
How the data were acquired	*Smartphone sensor data was captured while driver was executing the driving events in a realistic traffic condition. An Android application (Sensor Record) was used to record the parameters. The experiment was conducted for 7-days, and 2-trips per day. The drivers aged between 30 and 35 years, and having 5+ years of driving experience executed the driving events in realistic traffic situation.*
Data format	*Raw*
Description of data collection	*The” Sensor Record” mobile app was used to record the data from the Smartphone sensors. Each trip's recorded parameters were saved in a day- by-day .csv file. For each one-way travel, the dataset is divided into seven subfolders. Each folder also has .csv files for each sensor. The data was collected in a realistic traffic situation and under a variety of environmental circumstances. The proposed dataset can be used for driver behavior categorization or reorganization model training, testing, and validation.*
Data source location	*VISHWAKARMA UNIVERSITY, Survey No. 2, 3, 4 Laxmi Nagar, Kondhwa Budruk, Pune - 411 048. Maharashtra, India. Latitude and longitude: 18.4603 °N, 73.8836 °E* *CERATEC CITY, Kamthe Nagar, Yewalewadi, Pune – 411 048. Maharash tra, India. Latitude and longitude: 18.442866 °N, 73.884894 °E*
Data accessibility	Repository name: Driver Behavior Detection Using Smartphone - Dataset Data identification number: 10.17632/9vr83n7z5j.1 Direct URL to data: https://data.mendeley.com/datasets/9vr83n7z5j/2

## Value of the Data

• To simulate the driver behavior, data from Smartphone sensors (accelerometers, gyroscopes, etc.) was extracted by simulating real-world driving occurrences.
• Driving activities took place in a variety of road and traffic circumstances.
• Various vehicles (4-wheelers) and mobile phones were employed to carry out the events and collect data.
• The data set contains roughly 15,000+ sensor values for a one-way travel of 5–17 kms.
• The dataset is useful to build driver behavior classification with quality applications which are beneficial for improving driver safety, avoiding road accidents, reducing fuel consumption, and automotive industries as a whole.
• The dataset is useful in road accident analysis and prevention, by monitoring the driver's behavior behind the wheels.
• The dataset will be useful for training, testing and validation of driver behavior classification or reorganization model.
• If you can measure a driver's driving style, you can take a variety of steps to increase driver safety, road safety, fuel efficiency, and emissions.

## 1 Data Description

Dataset for driver behavior classification (normal, aggressive, risky) based on accelerometer (X, Y, Z axis in meters per second squared (m/s2)) and gyroscope (X, Y, Z axis in degrees per second (°/s)) data.

The data from the Smartphone sensors was recorded using the ``Sensor Record'' mobile app. The recorded parameters for each one-way trip were saved in a day-by-day folder, with.csv files for each individual sensor in each folder (Accelerometer.csv, Gyroscope.csv, etc.).Table 1 and Table 2: show the raw data samples from the accelerometer and gyroscope, respectively: (data was recorded with default settings of the Android app. For ex: timestamp indicates date and time of journey) Table 3: displays a folder description of the data collected for several drivers, whereas Table 4: shows a folder description of the data collected for a driver who completed driving events on different days.

**Table 1:** Accelerometer data.

Timestamp	Unix Timestamp	Milliseconds	X	Y	Z
02–02–21 10:28:14 AM	1,612,261,680	1	−1.63639	−0.60269	9.899107
02–02–21 10:28:14 AM	1,612,261,680	12	−1.69412	−0.49502	9.731311
02–02–21 10:28:14 AM	1,612,261,680	21	−1.64596	−0.63858	9.702597
02–02–21 10:28:14 AM	1,612,261,680	30	−1.74915	−0.55005	9.968499
02–02–21 10:28:14 AM	1,612,261,680	41	−1.66271	−0.45912	9.853643

**Table 2:** Gyroscope data.

Timestamp	Unix Timestamp	Milliseconds	X	Y	Z
02–02–21 10:28:14 AM	1,612,261,680	1	0.003595	−0.00426	−0.0028
02–02–21 10:28:14 AM	1,612,261,680	12	−6.66E-04	−0.00213	−6.66E-04
02–02–21 10:28:14 AM	1,612,261,680	20	0.001465	−0.0032	3.99E-04
02–02–21 10:28:14 AM	1,612,261,680	31	0.003595	−0.00213	−0.00173
02–02–21 10:28:14 AM	1,612,261,680	41	0.00466	−0.00213	−6.66E-04

**Table 3:** Data captured for various drivers.

Folder name	Date	Start time	End time
Driver-1	02–02–21	10:28:00 am	10:44:00 am
Driver-2	02–02–21	10:45:00 am	10:54:00 am
Driver-3	02–02–21	01:34:00 pm	01:52:00 pm
Driver-4	03–02–21	10:18:00 am	10:48:00 am
Driver-5	03–02–21	01:48:00 pm	02:03:00 pm
Driver-6	04–02–21	10:38:56 am	10:48:04 am
Driver-7	04–02–21	02:06:09 pm	02:23:28 pm

**Table 4:** Data captured for 2-way trip.

Folder name	Date	Start trip time	Return trip time
Day-1S/1R	23–05–2020	7:46 am	13:17 pm
Day-2S/2R	24–05–2020	7:53 am	12:20 pm
Day-3S/3R	25–05–2020	8:01 am	18:59 pm
Day-4S/4R	26–05–2020	7:56 am	13:06 pm
Day-5S/5R	28–05–2020	7:52 am	–
Day-6S/6R	29–05–2020	7:57 am	12:50 pm
Day-7S/7R	30–05–2020	8:00 am	–

**Table 5:** Experimental setup.

**Data gathering**	*Android application - Sensor Record (ver. 2.3.0) used for recording Smart-* *phone sensor data*
**Smartphone sensor**	*Accelerometer, Gyroscope.*
**Vehicle type**	*LMV*
**Vehicle models**	*Ford Figo 1.2, Maruti Suzuki Swift VXI, Tata Nexon XMS*
**Phone model**	*Redmi 4 with Android version 7.1.2*
**Phone position**	*Horizontally fixed in the car's utility box*
**Sampling rate**	*50* *Hz default*
**Driving experience**	*5+ years*
**Weather condition**	*Sunny*

Accurate driver behavior classification is critical in the fast-growing automotive and transportation industries [4]. Using computer vision and deep learning algorithms, driver behavior can be divided into distinct classifications based on their driving habits, such as normal, aggressive, and rash driving [5]. By automatically collecting driving data, we were able to generate a dataset (e.g., speed, acceleration, breaking, steering, and location). We were able to accomplish this by executing a data-gathering phase while driving a car and collecting data from a variety of sensors accelerometer, gyroscope, magnetometer, GPS, and so on.

Dynamic Temporal Warping (DTW), Threshold-based approaches, and machine learning methods [3] are the three basic approaches to Smartphone-based driver behavior classification [6]. Within Smartphone-based driver behavior classification, accelerometers stand out as the most commonly used sensor. We may use accelerometer data to perform end-point detection to determine when an event of interest has occurred. The event is then characterized as a normal or aggressive turn, acceleration, or braking using various MLAs or predetermined templates for each class.

## 2 Experimental Design, Materials and Methods

### 2.1 Experimental design

Smartphones include a variety of on-board sensors, like as accelerometers, gyroscopes, GPSs, and cameras, that can give useful data for analyzing driver behavior patterns and collecting vehicle data, which is then sent to a remote server for real-time and offline analysis [1,2]. In mobile phones, accelerometers are used to determine the phone's orientation. The linear acceleration of movement is measured by an accelerometer, whereas the angular rotational velocity is measured by a gyro. Both sensors measure rate of change; the difference is that they measure distinct things.

The next step is to create the experiment and the experimental conditions once the model driver (persona) has been finalized. We ran the experiment for seven days, with the driver making two journeys each day. Each one-way trip was around 17.2 kms long and took an average of 25 min. We utilized an Android application to record data from smart phone sensors (accelerometer, gyroscope) in this experiment. While the driver was conducting the driving activities, the smart phone was horizontally fixed on the car's utility box. The experimental setup is detailed in Table 5.

### 2.2 Materials or specification of data acquisition system

The driving behavior dataset was acquired by placing the Redmi 4 Smartphone horizontally in the vehicle's utility box and using its sensors. All .csv files from the dataset are saved in a day-to-day folder. Data was collected in a range of realistic traffic scenarios with various cars and drivers. To comprehend the driver's behavior, we must investigate the other aspects that influence his or her driving performance [7]. One of the factors that must be examined for driver behavior classification is internal variables, or the in-car environment. Inner variables such as driving setting, secondary tasks completed, phone usage, emotional status, and so on are taken into account as factors influencing driver behavior when executing driving events in our situation. We were able classify the driving behavior into normal/aggressive/risky driving category after applying different MLAs on the dataset [3].

## Ethics Statements

There is no funding for the present effort. There is no conflict of interest. The data is available in public domain.

Informed consent was obtained from the drivers involved in executing the driving events for this data acquisition.

## Data Availability

Driver behavior detection using smartphone - dataset (Original data) (Mendeley Data).

## CRediT authorship contribution statement

**Pawan Wawage:** Methodology, Software, Validation, Writing – original draft. **Yogesh Deshpande:** Conceptualization, Supervision, Writing – review & editing.

## Declaration of Competing Interest

The authors declare that they have no known competing financial interests or personal relationships that could have appeared to influence the work reported in this paper.
